# Robot-Embodied Neuronal Networks as an Interactive Model of Learning

**DOI:** 10.2174/1874205X01711010059

**Published:** 2017-12-04

**Authors:** Abraham M Shultz, Sangmook Lee, Mary Guaraldi, Thomas B. Shea, Holly A. Yanco

**Affiliations:** 1 Robotics Laboratory, Department of Computer Science, USA; 2 Laboratory for Neuroscience, Department of Biological Sciences University of Massachusetts Lowell, Lowell, MA 01854, USA

The correct names are provided and replaced online which is mentioned as under:

Abraham M Shultz, Sangmook Lee, Mary Guaraldi, Thomas B. Shea, Holly A. Yanco

The original names was:

Abraham M Shultz, Sangmook Lee, Mary Guaraldi, Thomas B. Shea, Holly C. Yanco

